# Preterm delivery and risk of breast cancer

**DOI:** 10.1038/sj.bjc.6690399

**Published:** 1999-05-01

**Authors:** M Melbye, J Wohlfahrt, A-M N Andersen, T Westergaard, P K Andersen

**Affiliations:** Danish Epidemiology Science Centre, Statens Serum Institut, 5 Artillerivej, DK-2300, Copenhagen S, Denmark

**Keywords:** breast cancer, reproductive factors, gestational age, preterm, cohort study, population-based

## Abstract

To explore the risk of breast cancer in relation to the length of a pregnancy we tested whether a preterm delivery carries a higher risk of breast cancer than does a full-term delivery. Based on information from the Civil Registration System, and the National Birth Registry in Denmark, we established a population-based cohort of 474 156 women born since April 1935, with vital status and detailed parity information, including the gestational age of liveborn children and stillbirths. Information on spontaneous and induced abortions was obtained from the National Hospital Discharge Registry and the National Registry of Induced Abortions. Incident cases of breast cancer in the cohort (*n* = 1363) were identified through linkage with the Danish Cancer Registry. The period at risk started in 1978 and continued until a breast cancer diagnosis, death, emigration, or 31 December, 1992, whichever occurred first. After adjusting for attained age, parity, age at first birth and calendar period, we observed the following relative risks of breast cancer for different lengths of the pregnancy: < 29 gestational weeks = 2.11 (95% confidence interval 1.00–4.45); 29–31 weeks = 2.08 (1.20–3.60); 32–33 weeks = 1.12 (0.62–2.04); 34–35 weeks = 1.08 (0.71–1.66); 36–37 weeks = 1.04 (0.83–1.32); 38–39 weeks = 1.02 (0.89–1.17); 40 weeks = 1 (reference). Parous women who had a preterm delivery below 32 weeks gestation had a 1.72-fold (1.14–2.59) increased risk of breast cancer compared with other parous women. In conclusion, a preterm delivery of 32+ weeks gestation did not significantly increase a woman's risk of contracting breast cancer. Only for the very small group of women with preterm deliveries of less than 32 weeks gestation did we observe an increased risk. © 1999 Cancer Research Campaign


*British Journal of Cancer* (1999) **80**(3/4), 609–613
© 1999 Cancer Research Campaign
Article no. bjoc.1998.0399

### Preterm delivery and risk of breast cancer

**M Melbye, J Wohlfahrt, A-MN Andersen, T Westergaard and PK Andersen**

Danish Epidemiology Science Centre, Statens Serum Institut, 5 Artillerivej, DK-2300 Copenhagen S, Denmark

**Summary** To explore the risk of breast cancer in relation to the length of a pregnancy we tested whether a preterm delivery carries a higher risk of breast cancer than does a full-term delivery. Based on information from the Civil Registration System, and the National Birth Registry in Denmark, we established a population-based cohort of 474 156 women born since April 1935, with vital status and detailed parity information, including the gestational age of liveborn children and stillbirths. Information on spontaneous and induced abortions was obtained from the National Hospital Discharge Registry and the National Registry of Induced Abortions. Incident cases of breast cancer in the cohort ($n$ = 1363) were identified through linkage with the Danish Cancer Registry. The period at risk started in 1978 and continued until a breast cancer diagnosis, death, emigration, or 31 December, 1992, whichever occurred first. After adjusting for attained age, parity, age at first birth and calendar period, we observed the following relative risks of breast cancer for different lengths of the pregnancy: < 29 gestational weeks = 2.11 (95% confidence interval 1.00–4.45); 29–31 weeks = 2.08 (1.20–3.60); 32–33 weeks = 1.12 (0.62–2.04); 34–35 weeks = 1.08 (0.71–1.66); 36–37 weeks = 1.04 (0.83–1.32); 38–39 weeks = 1.02 (0.89–1.17); 40 weeks = 1 (reference). Parous women who had a preterm delivery below 32 weeks gestation had a 1.72-fold (1.14–2.59) increased risk of breast cancer compared with other parous women. In conclusion, a preterm delivery of 32+ weeks gestation did not significantly increase a woman's risk of contracting breast cancer. Only for the very small group of women with preterm deliveries of less than 32 weeks gestation did we observe an increased risk.

**Keywords:** breast cancer; reproductive factors; gestational age; preterm; cohort study; population-based

Major hormones influence the development, proliferation and differentiation of the human breast (Rebar, 1994). Based primarily on animal studies, it has been shown that mammary cells proliferate in the first and second trimester of pregnancy and differentiate in the last trimester (Russo and Russo, 1980). This led Russo and Russo to hypothesize that complete differentiation of the breast cells conveyed by a full-term pregnancy has to be achieved to provide protection against carcinogenic effects. Earlier termination of pregnancy, on the contrary, might increase the risk of breast cancer because proliferation of the breast cells will take place without subsequent differentiation (Russo and Russo, 1980).

Breast cancer risk in women with a history of a short-term pregnancy has primarily been investigated in relation to spontaneous and induced abortions (Kvåle et al, 1987; Adami et al, 1990; Daling et al, 1994; Calle et al, 1995; Michels et al, 1995; Newcomb et al, 1996; Melbye et al, 1997) that occur during the early period of pregnancy. In particular, large prospective studies have not found such women to be at increased risk of breast cancer (Kvåle et al, 1987; Calle et al, 1995; Melbye et al, 1997). In contrast, few studies have addressed the late period of pregnancy and whether a preterm delivery is associated with an increased risk of breast cancer (Choi et al, 1978; Polednak and Janerich, 1983).

In the present study we took advantage of the long tradition for mandatory reporting of pregnancy characteristics and cancer diagnoses in Denmark to address in a prospective study whether women with preterm delivery are at increased risk of breast cancer compared to other women.

*Received 28 July 1998*
*Revised 5 October 1998*
*Accepted 21 October 1998*

*Correspondence to:* M Melbye

### MATERIAL AND METHODS

### Registries

We performed a linkage of data from the Danish Civil Registration System (CRS) with the National Birth Registry, the National Hospital Discharge Registry, the National Registry of Induced Abortions and the Danish Cancer Registry. Since April 1968, the CRS has assigned a unique identification number to all residents in Denmark which permits accurate linkage of information from different registries. The CRS also keeps updated information on dates of live births and documents demographic information such as emigration and death.

Since 1973 the National Birth Registry has registered all livebirths and stillbirths in Denmark (not including spontaneous and induced abortions). Since 1978, exact (in weeks) gestational age determinations have been included. Gestational age determination is based on information of last menstrual period combined with an early clinical bimanual palpation. In situations of inconsistency between these measures, ultrasound scanning is performed. In the most recent years the use of ultrasound scanning has become widespread and has as such contributed increasingly to the determinations of the gestational age (Sundhedsstyrelsen, 1993). Since 1977, information on spontaneous abortions without specified gestational age has been recorded in the National Hospital Discharge Registry. Information on induced abortions has been recorded in the National Registry of Induced Abortions since reporting became mandatory in 1939. However, information is only available in a computerized format since 1973 (Melbye et al,

1997). The Danish Cancer Registry includes a nearly complete registration of cancer diagnoses on all Danish residents back to 1943 (Storm, 1991).

### Subjects

A research database was established from the CRS including all women born in Denmark between 1 April 1935 and 31 March 1978, with information on live-born children. From the National Birth Registry additional information on stillbirths was added as was gestational age-specific information on all births since 1978. Finally, information on spontaneous (since 1977) and induced abortions (since 1973) was added.

### Analyses

The possible impact of gestational age at delivery (preterm, or term delivery) on the risk of breast cancer was investigated among parous women in a log-linear Poisson regression model (Breslow and Day, 1987). All women entered the follow-up for breast cancer at the first delivery they had during the period between 1 January 1978 and 31 December 1992, in which gestational age was recorded. Thus, women with pregnancies before 1 January 1978 were included in the study provided they had a delivery during the study period. The period at risk continued until breast cancer diagnosis, death, emigration, disappearance, or 31 December 1992 (at which time the cancer registration was considered complete), whichever occurred first. Person-years at risk were calculated continuously according to the categorical groups of gestational age of the most recent birth in the years 1978–1992, i.e. women with more than one birth between 1978 and 1992 were considered at risk in the period between the first and second birth, according to the gestational age of the first birth; between the second and third birth, according to the gestational age of the second birth; and so on. To evaluate the effect of *ever* having a preterm delivery, an additional analysis was performed where person-years at risk were calculated continuously in categorical groups according to the birth with the lowest gestational age since 1978. Adjustments were made for attained age (1-year intervals), calendar period (5-year intervals), age at first birth (12–19, 20–24, 25–29, 30–34, > 34 years) and parity (1, 2, 3, 4, 5, 6, ≥ 7 births; including stillbirths, preterm and term deliveries). In an additional analysis we adjusted for history of spontaneous and induced abortion and whether the birth was a stillbirth or a multiple birth. Note that information on history of spontaneous and induced abortions, stillbirths and live-births prior to 1 January 1978 was also used in the adjustment. Estimation of breast cancer incidence rate ratios was performed using the SAS procedure PROC GENMOD (SAS Institute, 1996). These rate ratios were used as a measure of the relative risk (RR). Test for trend was performed with gestational age treated as a continuous variable and the median gestational age used as the value for each group. The linear assumption in the trend test was checked by a likelihood ratio test against the model with gestational age as categorical variable. Effect modification was evaluated as a test for interaction between categorical variables.

To assess the possible effect of misclassification due to unregistered gestational age in births prior to 1978 we estimated the percentage of person-years of follow-up and the number of cases in each cell that might be attributed to the ‘ever had a delivery with a gestational age less than 32 weeks’ category, instead of the ‘never’ category, and then performed the analysis with the adjusted figures. The percentage of person-years was calculated on the basis of the age-specific cumulative incidence at the baseline of the study, and the number of cases was calculated as the product of the estimated person-years and the rate in the ever category found in the original analysis. The age-specific cumulative incidence of having a delivery with a gestational age less than 32 weeks was calculated using age-specific incidence rates seen in 1983–1992.

### RESULTS

Overall, 474 156 parous women were included in the cohort study. In the follow-up a total of 740 794 births were recorded and distributed as follows: 254 458 women (53.7%) had one birth, 178 700 women (37.7%) had two, 35 791 women (7.5%) had three and 5207 women (1.1%) had four or more births. Among these births, 3261 were stillbirths (0.4%) and 37 347 (5.0%) were preterm (< 37 gestational weeks). Preterm births with a gestational age of 32–36 weeks contributed 4.2%, with a gestational age of

**Table 1** Distribution of number of breast cancer diagnoses and person-years of follow-up according to age and reproductive history

| | preterm delivery | | | | Full-term delivery | | | |
|---|---|---|---|---|---|---|---|---|
| | No. of cases | (%) | Person-years ($\times 10^3$) | (%) | No. of cases | (%) | Person years ($\times 10^3$) | (%) |
| Age (years) | | | | | | | | |
| < 35 | 16 | (20) | 127 | (69) | 315 | (25) | 2507 | (70) |
| 35–39 | 31 | (38) | 35 | (19) | 417 | (32) | 714 | (20) |
| 40–44 | 24 | (30) | 16 | (9) | 379 | (30) | 299 | (8) |
| 45–49 | 8 | (10) | 5 | (3) | 147 | (11) | 72 | (2) |
| 50+ | 2 | (2) | 1 | (0.4) | 24 | (2) | 9 | (0.2) |
| Age at first birth (years) | | | | | | | | |
| < 20 | 9 | (11) | 30 | (17) | 93 | (7) | 464 | (13) |
| 20–24 | 24 | (30) | 82 | (45) | 432 | (34) | 1728 | (48) |
| 25–29 | 27 | (33) | 52 | (28) | 501 | (39) | 1107 | (31) |
| 30–34 | 18 | (22) | 15 | (8) | 191 | (25) | 254 | (7) |
| 35+ | 3 | (4) | 4 | (2) | 65 | (5) | 48 | (1) |
| Age at latest birth (years) | | | | | | | | |
| < 20 | 0 | (0) | 8 | (4) | 1 | (0.1) | 105 | (3) |
| 20–24 | 1 | (1) | 47 | (26) | 54 | (4) | 874 | (24) |
| 25–29 | 23 | (28) | 68 | (37) | 351 | (28) | 1449 | (40) |
| 30–34 | 29 | (36) | 41 | (22) | 513 | (40) | 872 | (24) |
| 35+ | 28 | (35) | 20 | (11) | 363 | (28) | 300 | (9) |
| Number of previous births[a] | | | | | | | | |
| 0 | 23 | (28) | 78 | (42) | 240 | (19) | 1281 | (36) |
| 1 | 31 | (38) | 68 | (37) | 611 | (48) | 1609 | (45) |
| 2 | 19 | (24) | 27 | (15) | 313 | (24) | 553 | (15) |
| 3+ | 8 | (10) | 11 | (6) | 118 | (9) | 157 | (4) |
| Previous preterm birth or stillbirth[a] | | | | | | | | |
| Yes | 5 | (6) | 12 | (7) | 17 | (1) | 60 | (2) |
| No | 76 | (94) | 171 | (93) | 1265 | (99) | 3540 | (98) |
| The delivery was a multiple birth | | | | | | | | |
| Yes | 9 | (11) | 16 | (9) | 20 | (2) | 35 | (1) |
| No | 72 | (89) | 167 | (91) | 1262 | (98) | 3566 | (99) |

[a] ‘Previous’ means prior to the most recent pregnancy.

*© Cancer Research Campaign 1999*

**Table 2** Adjusted[a] relative risk of breast cancer in 474 156 parous women according to gestational age at delivery

| Gestational age (weeks) | No. of cases | Person-years (× 10³) | RR (95% CI) |
|---|---|---|---|
| < 29 | 7 | 9 | 2.11 (1.00–4.45) |
| 29–31 | 13 | 17 | 2.08 (1.20–3.60) |
| 32–33 | 11 | 26 | 1.12 (0.62–2.04) |
| 34–35 | 22 | 58 | 1.08 (0.71–1.66) |
| 36–37 | 82 | 214 | 1.04 (0.83–1.32) |
| 38–39 | 350 | 949 | 1.02 (0.89–1.17) |
| 40 | 552 | 1526 | 1 |
| > 40 | 326 | 985 | 1.03 (0.90–1.18) |

[a]Adjusted for age, calendar period, parity and age at first birth.

29–31 weeks 0.5%, and with a gestational age of less than 29 weeks 0.3%. The number of women with a preterm delivery was as follows: 32–36 weeks = 29 488 women; 29–31 weeks = 3702 women; < 29 weeks = 2181 women. Parous women represented a total of 3.8 million person-years of follow-up and 1363 of these women developed breast cancer. Table 1 presents a detailed distribution of number of breast cancer diagnoses and person-years of follow-up.

As shown in Table 2, we found a significantly increased relative risk of breast cancer in women with a preterm delivery at < 29 gestational weeks of 2.11 (95% confidence intervals (CI) 1.00–4.45) and at 29–31 gestational weeks of 2.08 (1.20–3.60), which subsequently dropped as follows: 32–33 weeks: RR = 1.12 (0.62–2.04); 34–35 weeks: RR = 1.08 (0.71–1.66); 36–37 weeks: RR = 1.04 (0.83–1.32); 38–39 weeks: RR = 1.02 (0.89–1.17), 40 weeks: 1 (reference). The continued decline in RR observed for preterm deliveries was statistically significant ($P$-trend = 0.04). The trend remained significant after adjustment for history of spontaneous abortion, history of induced abortion, and whether the birth was a stillbirth and/or a multiple birth ($P$-trend = 0.04). A stratified analysis, which was performed to evaluate whether the increased risk of breast cancer was associated both with preterm livebirths and preterm stillbirths, gave the following result with term deliveries as reference: life births with gestational age < 32 weeks: RR = 1.98 (1.24–3.16); stillbirths with gestational age < 32 weeks: RR = 4.62 (0.42–50.9).

The possible effect modification by age of the woman, number of previous births, age at delivery and history of previous preterm births or stillbirths is evaluated in Table 3. None of these characteristics significantly modified the risk association observed with gestational age. However, the number of cases in some of the stratified subgroups became very small. We evaluated whether possible temporal changes in the validity and completeness of the ascertainment of the gestational age had a measurable effect on the results by testing whether there was a significant effect modification by period of delivery. This was not the case ($P$ = 0.62).

Comparing parous women *ever* having a delivery of less than 32 gestational weeks with other parous women we found a significantly increased risk of 1.72 (1.14–2.59). When we considered only parous women ever having a delivery less than 32 weeks' gestation, but with the most recent delivery being equal to or longer than 32 weeks' gestation, we found no increased risk when comparing with parous women who had never had a delivery of less than 32 gestational weeks (RR = 0.82; 95% CI: 0.26–2.55). However, this result was based on only three cases of breast cancer in this particular group of women.

Based on the age-specific incidence rates of births with a gestational age less than 32 weeks we estimated that less than 2% will ever experience such a delivery. Taking that into account at the baseline of the analysis the rate ratio between parous women ever

**Table 3** Adjusted[a] relative risk of breast cancer in parous women according to gestational age at delivery by age, number of previous births, age at delivery and history of preterm births/stillbirths

| | Gestational age | | | | | |
|---|---|---|---|---|---|---|
| | ≥ 37 weeks | | 36–32 weeks | | < 32 weeks | |
| | No. of cases | RR (ref.) | No. of cases | RR (95% CI) | No. of cases | RR (95% CI) |
| Age of woman[b] | | | | | | |
| < 40 years | 732 | 1 | 37 | 1.21 (0.87–1.69) | 10 | 2.00 (1.07–3.74) |
| ≥ 40 years | 550 | 1 | 24 | 0.88 (0.58–1.32) | 10 | 2.11 (1.13–3.95) |
| Number of previous[c] births[d] | | | | | | |
| 0 | 240 | 1 | 17 | 1.14 (0.70–1.87) | 6 | 2.41 (1.07–5.42) |
| 1+ | 1042 | 1 | 44 | 1.03 (0.76–1.39) | 14 | 1.94 (1.14–3.29) |
| Age at delivery[e] | | | | | | |
| < 30 years | 406 | 1 | 20 | 1.20 (0.77–1.89) | 4 | 1.62 (0.60–4.33) |
| ≥ 30 years | 876 | 1 | 41 | 1.00 (0.73–1.37) | 16 | 2.22 (1.35–3.64) |
| Previous[6] preterm birth[f] or stillbirth[g] | | | | | | |
| No | 1265 | 1 | 58 | 1.06 (0.82–1.38) | 18 | 1.97 (1.24–3.14) |
| Yes | 17 | 1 | 3 | 1.02 (0.30–3.49) | 2 | 3.64 (0.84–15.8) |

[a]Adjusted for age of the woman, calendar period, parity and age at first birth. [b]Test for effect modification: $P$ = 0.47. A similar lack of effect modification ($P$ = 0.73) was found if age of woman was divided by age 50 years. [c]'Previous' means prior to the most recent pregnancy. [d]Test for effect modification: $P$ = 0.86. [e]Test for effect modification: $P$ = 0.67. [f]Pre-term birth: gestational age < 37 weeks. [g]Test for effect modification: $P$ = 0.76.

© Cancer Research Campaign 1999

having a delivery less than 32 gestational weeks and other women increased from 1.72 to 1.73.

### DISCUSSION

Based on this large cohort of almost half a million parous women we found reassuring evidence that a preterm delivery of 32+ weeks' gestation does not significantly increase the risk of premenopausal breast cancer. Overall, 84% of all preterm deliveries are of 32+ weeks' gestation. Only for the small group of preterm deliveries of less than 32 weeks' gestation was there a twofold increased risk of breast cancer when comparing with a full-term delivery. This elevated relative risk was obtained in an analysis in which a woman's person-years at risk were calculated continuously according to the gestational age of the most recent birth. In an analysis that compared parous women ever having a delivery of less than 32 gestational weeks with other parous women the risk was 1.7-fold increased. In this last analysis, the preterm birth will not necessarily have been the most recent birth, and we speculate whether the somewhat lower estimate could indicate that a full-term birth following a preterm birth might diminish the effect of a preterm birth on breast cancer risk. We found some support for this assumption in a restricted analysis that estimated the risk in parous women ever having a delivery of less than 32 weeks' gestation but with the most recent delivery being of 32+ gestational weeks. However, this particular analysis has very limited power.

The analysis of parous women *ever* having a delivery with a gestational age less than 32 weeks compared with other women might be subject to some misclassification, since many of the included women may have had preterm births prior to 1978. This misclassification, however, is non-differential, and estimating the effect, we found we could ignore it, as only a very small fraction of women categorized as never having a delivery with a gestational age less than 32 weeks in fact had such a birth prior to 1978.

We used a cohort design for our study based on mandatory reported exposure and outcome information. Nonetheless, some limitations of the study should be acknowledged. Our gestational age-specific RR estimates do not follow a smooth curve, but instead increase rather abruptly below 32 weeks' gestation. This might suggest that the elevated risk of breast cancer among women with a very early preterm delivery was a chance finding. However, another explanation would be that the small number of cases with very early preterm deliveries makes it difficult to assess the true magnitude of the effect. In particular, the estimate obtained among women with a preterm delivery of less than 29 weeks was based on only seven cases of breast cancer and 9000 person-years of follow-up. That said, it is important to note that this estimate did not stand alone but was supported by a similarly increased risk for women with a preterm delivery of 29–31 gestational weeks. We were unable to determine whether the observed risk was due to the preterm delivery per se or the shorter duration of pregnancy. The observation that both women with a preterm stillbirth and women with a preterm livebirth (< 32 weeks) had elevated RR of breast cancer would be in support of the latter but these were very few.

The present study allowed us to consider the influence of potentially confounding factors such as age, age at first birth, parity, multiple births, abortion history and history of stillbirths. However, several factors (smoking history, body mass index, age at menarche and menopause, family history, oral contraceptives, postmenopausal hormones) that have been suspected as risk factors for breast cancer could not be adjusted for because we lacked the necessary information. The lack of adjustment for such factors would only be important for our results should these factors influence both the occurrence of breast cancer and preterm births. Smoking during pregnancy and high pre-pregnant body weight have been linked to preterm births (Naeye, 1990; Williams et al, 1992). However, there is little evidence for an association between smoking and breast cancer (Palmer and Rosenberg, 1993) and the association between high body mass and premenopausal breast cancer is, if anything, inverse (Hunter and Willett, 1993). Other factors that have been associated with preterm births are low social class and low educational level (Pickering and Deeks, 1991). However, breast cancer risk is associated with high social status and thus we would expect the observed relative risks to be underestimated, rather than the opposite.

We are not aware of any previous cohort study addressing the risk of breast cancer according to week of gestation at delivery. In a case-control study, Choi et al (1978) reported an insignificantly 1.4-fold increased risk of breast cancer in premenopausal women who had a terminated pregnancy of more than 5 gestational months compared to women without such experience. Another case-control study focusing on livebirths, with seven women with a delivery of less than 30 weeks, did not find an increased risk among women with preterm deliveries (Polednak and Janerich, 1983). Stillbirth has not been associated with increased risk of breast cancer, but the available studies have been based on a very limited number of cases and lacked information on gestational length of the pregnancy (Brimton et al, 1983; Rao et al, 1994; Calle et al, 1995).

Studies of spontaneous abortion have generally not revealed significantly positive associations (reviewed in Calle et al, 1995). In a recent study by Newcomb et al (1996), a slightly increased risk of breast cancer was recorded, but the authors cautioned that the finding might be due to recall bias in their case-control design. Most spontaneous abortions take place early in pregnancy and studies have so far lacked detailed information on gestational week at the time of the abortion. Spontaneous abortion may in certain ways be more like a preterm delivery than an induced abortion but they both represent an interruption of pregnancy (Zang, 1996). The results of case-control studies on induced abortion have been inconsistent with risk estimates ranging from moderately elevated to lowered values (Rosenberg et al, 1994). In a large prospective study we found no overall increased risk of breast cancer after an induced abortion, with the exception of the very small group of women with a late second trimester abortion (Melbye et al, 1997).

In conclusion, a preterm delivery did not significantly increase a woman's risk of contracting premenopausal breast cancer, apart from the very small group of women with a preterm delivery of less than 32 weeks' gestation. Despite the large size of this study there were only a few cases of breast cancer in the subgroups representing the very early deliveries and these results should therefore be considered with due caution.

### ACKNOWLEDGEMENTS

This study was supported by the US Army Breast Cancer Research Program (DAMD179616321) and the Danish National Research Foundation.

*© Cancer Research Campaign 1999*

### REFERENCES

Adami HO, Bergstøm E, Lund E and Meirik O (1990) Absence of association between reproductive variables and the risk of breast cancer in young women in Sweden and Norway. *Br J Cancer* **62**: 122–126

Breslow NE and Day NE (1987) *Statistical Methods in Cancer Research. Vol. 2, The Design and Analysis of Cohort Studies*. IARC Scientific Publications No. 82. IARC: Lyon

Brinton LA, Hoover R and Fraumeni JF (1983) Reproductive factors in the aetiology of breast cancer. *Br J Cancer* **47**: 757–762

Calle EE, Mervis CA, Wingo PA, Thun MJ, Rodriguez C and Health CW (1995) Spontaneous abortion and risk of fatal breast cancer in a prospective cohort of United States women. *Cancer Causes Control* **6**: 460–468

Choi NW, Howe GR, Miller AB, Matthews V, Morgan RW, Munan L, Burch JD, Feather J, Jain M and Kelly A (1978). An epidemiologic study of breast cancer. *Am J Epidemiol* **107**: 510–521

Daling JR, Malone KE, Voight LF, White E and Weiss NS (1994) Risk of breast cancer among young women: relationship to induced abortion. *J Natl Cancer Inst* **86**: 1584–1592

Hunter DJ and Willett WC (1993) Diet, body size and breast cancer. *Epidemiology Rev* **15**: 110–132

Kvåle G, Heuch I and Eide GE (1987) A prospective study of reproductive factors and breast cancer. I. Parity. *Am J Epidemiol* **126**: 831–841

Melbye M, Wohlfahrt J, Olsen JH, Frisch M, Westergaard T, Helweg-Larsen K and Andersen PK (1997) Induced abortion and the risk of breast cancer. *N Engl J Med* **336**: 81–85

Michels KB, Hsieh CC, Trichopoulos D and Willett WC (1995) Abortion and breast cancer risk in seven countries. *Cancer Causes Control* **6**: 75–82

Naeye R (1990) Maternal body weight and pregnancy outcome. *Am J Clin Nutr* **52**: 273–279

Newcomb PA, Storer BE, Longnecker MP, Mittendorf R, Greenberg ER and Willett WC (1996) Pregnancy termination in relation to risk of breast cancer. *JAMA* **275**: 283–287

Palmer JR and Rosenberg L (1993) Cigarette smoking and the risk of breast cancer. *Epidemiol Rev* **15**: 145–156

Pickering RM and Deeks JJ (1991) Risks of delivery during the 20th to 36th week of gestation. *Int J Epidemiol* **20**: 456–466

Polednak AP and Janerich DT (1983) Characteristics of first pregnancy in relation to early breast cancer. A case-control study. *J Reprod Med* **28**: 314–318

Rao DN, Ganesh B and Desai PB (1994) Role of reproductive factors in breast cancer in a low-risk area: a case-control study. *Br J Cancer* **70**: 129–132

Rebar RW (1994) The breast and the physiology of prolactation. In: *Maternal Fetal Medicine*: *Principles and Practice*, Creasy RK and Resnik R (eds), pp. 144–161. WB Saunders: Philadelphia

Rosenberg L (1994) Induced abortion and breast cancer: more scientific data are needed. *J Natl Cancer Inst* **86**: 1569–1570

Russo J and Russo IH (1980) Susceptibility of the mammary gland to carcinogenesis. II Pregnancy interruption as a risk factor in tumor incidence. *Am J Pathol* **100**: 497–512

SAS Institute Inc. (1996) SAS/STAT® Software: Changes and Enhancements, Release 6.11, SAS Institute Inc: Cary, NC

Storm HH (1991) Appendix 3(a): the Danish Cancer Registry, a self-reporting national cancer registration system with elements of active data collection. In: *Cancer Registration*: *Principles and Methods*, Jensen OM, Parkin DM, MacLennan R, Muir CS and Skeet RG (eds), pp. 220–236. IARC Scientific Publications no. 95. IARC: Lyon

Sundhedsstyrelsen (1993) Statistik om praevention og aborter 1991 og 1992. *Vitalstatistik* **1**: 36

Williams MA, Mittendorf R, Stubblefield PG, Lieberman E, Schoenbaum SC and Monson RR (1992) Cigarettes, coffee, and premature rupture of the membranes. *Am J Epidemiol* **135**: 895–903

Zang J (1996) Differences between spontaneous and induced abortions as risk factors for breast cancer. *Epidemiology* **7**: 316–318

*© Cancer Research Campaign 1999*
